# Draft Genome Sequences of Enterobacter spp., *Lelliottia* spp., and *Serratia* spp., Coliform Bacteria from Drinking Water Reservoirs and Lakes

**DOI:** 10.1128/MRA.00622-21

**Published:** 2021-08-12

**Authors:** Carolin Reitter, Klaus Neuhaus, Michael Hügler

**Affiliations:** a TZW: DVGW-Technologiezentrum Wasser (German Water Center), Karlsruhe, Germany; b Core Facility Microbiome, ZIEL–Institute for Food & Health, Technical University of Munich, Freising, Germany; University of Maryland School of Medicine

## Abstract

Surface waters are a major source for drinking water production. Therefore, it is essential to examine microbial processes within the water bodies, such as mass proliferations of coliform bacteria. Here, we report the draft genome sequences of Enterobacter spp., *Lelliottia* spp., and *Serratia* spp. isolated from drinking water reservoirs and lakes.

## ANNOUNCEMENT

Worldwide, surface waters such as lakes and drinking water reservoirs are major sources for drinking water production. In recent years, high concentrations of coliform bacteria have been observed during the summer months, with Enterobacter spp. and *Lelliottia* spp. forming “coliform blooms,” whereas *Serratia* spp. dominate in winter (1–3). As coliform bacteria occasionally overcome treatment and reach drinking water, further research is needed with regard to their hygienic relevance and their capability to proliferate in these oligotrophic environments (3). Traditionally regarded as fecal indicator bacteria, coliform bacteria also occur naturally in water, plants, and soil (4–6). Toward this end, whole-genome shotgun sequencing of 21 coliform bacteria isolates was performed (Table 1).

**TABLE 1:** Characteristics and accession numbers of genomes from coliform bacteria isolated in drinking water reservoirs

Bacterial species^*a*^	Strain	Reservoir/lake	Read count (bp)	Genome coverage (×)	Genome size (bp)	No. of contigs	*N*_50_ (bp)	G+C content (%)	No. of CDSs^*b*^	GenBank accession no.	SRA no.
Enterobacter asburiae	TZW01	Klingenberg	5,949,546	370	4,940,293	45	355,075	55.3	4,782	JAEEAT000000000	SRR14430924
Enterobacter asburiae	TZW02	Klingenberg	5,088,393	317	4,661,786	26	454,690	55.7	4,386	JAENNF000000000	SRR14430923
Enterobacter asburiae	TZW03	Lake Constance	4,858,338	302	4,631,365	40	336,147	55.9	4,284	JAENNE000000000	SRR14430912
Enterobacter asburiae	TZW04	Wahnbach	5,719,871	356	4,800,941	19	396,606	55.6	4,589	JAENND000000000	SRR14430910
Enterobacter asburiae	TZW05	Klingenberg	5,527,150	344	4,747,844	40	231,680	55.6	4,478	JAENNC000000000	SRR14430909
Enterobacter asburiae	TZW06	Lake Constance	5,064,247	315	4,684,044	45	311,897	55.6	4,417	JAENNB000000000	SRR14430908
Enterobacter asburiae	TZW07	Lake Constance	5,322,862	331	4,795,094	25	502,428	55.7	4,555	JAENNA000000000	SRR14430907
Enterobacter asburiae	TZW08	Rappbode	5,225,148	325	4,792,810	33	470,802	55.6	4,547	JAENMZ000000000	SRR14430906
Enterobacter asburiae	TZW09	Obersee	5,305,554	330	4,746,220	36	323,238	55.6	4,468	JAENMY000000000	SRR14430905
Enterobacter asburiae	TZW10	Breitenbach	5,797,166	361	4,879,403	23	502,282	55.6	4,669	JAENMX000000000	SRR14430904
Enterobacter asburiae	TZW11	Wahnbach	5,071,083	315	4,803,492	19	396,606	55.6	4,592	JAENMW000000000	SRR14430922
Lelliottia amnigena	TZW12	Kleine Kinzig	6,333,162	428	4,694,183	26	415,957	52.5	4,382	JAENMV000000000	SRR14430921
Lelliottia amnigena	TZW13	Kleine Kinzig	5,906,285	399	4,830,285	26	337,333	52.5	4,577	JAENMU000000000	SRR14430920
Lelliottia amnigena	TZW14	Klingenberg	5,488,494	371	4,516,381	17	731,232	52.8	4,198	JAENMT000000000	SRR14430919
Lelliottia amnigena	TZW15	Söse	5,085,780	344	4,756,711	36	346,396	52.6	4,473	JAENMS000000000	SRR14430918
Lelliottia amnigena	TZW16	Söse	5,417,881	366	4,756,331	35	346,396	52.6	4,472	JAENMR000000000	SRR14430917
Lelliottia aquatilis	TZW17	Kleine Kinzig	5,684,773	360	4,835,026	27	233,298	53.8	4,472	JAENMQ000000000	SRR14430916
Serratia fonticola	TZW18	Kleine Kinzig	5,104,384	269	5,752,035	55	190,940	53.7	5,178	JAENMP000000000	SRR14430915
Serratia fonticola	TZW19	Kleine Kinzig	6,409,833	338	6,050,704	65	181,482	53.6	5,437	JAENMO000000000	SRR14430914
Serratia fonticola	TZW20	Schönbrunn	5,601,327	295	6,223,117	58	218,606	53.7	5,590	JAENMN000000000	SRR14430913
Serratia marcescens	TZW21	Kleine Kinzig	5,039,584	292	5,248,648	27	2,884,332	59.4	4,942	JAENMM000000000	SRR14430911

a Strain identification based on 1,000 single-copy genes using the Randomized Axelerated Maximum Likelihood (RAxML) tool in the codon tree pipeline at PATRIC.

b CDSs, coding DNA sequences.

Coliform bacteria were isolated from 2014 to 2019 during coliform mass proliferations from drinking water reservoirs and lakes in Germany using Colilert-18/Quanti-Tray tests (IDEXX Laboratories, Westbrook, ME, USA) according to ISO 9308-2 (7). To obtain single colonies, liquid from wells was transferred onto heterotrophic plate count agar plates (Merck KGaA, Darmstadt, Germany), recommended by German regulations (Deutsches Einheitsverfahren), and incubated for 24 h at 36°C. Bacterial isolates were picked and transferred to fresh heterotrophic plate count agar plates, where they were incubated under the same conditions as before. Genomic DNA of pure cultures, grown on these agar plates, was extracted using a FastDNA spin kit for soil (MP Biomedicals, Santa Ana, CA, USA) and quantified using a Qubit fluorometer (Invitrogen, Carlsbad, CA, USA) according to the manufacturer’s instructions.

Preparation of sequencing libraries was performed using a DNA prep kit (Illumina). The draft genomes were sequenced by 150-bp paired-end sequencing on an Illumina NextSeq 1000 instrument using NovaGene (Illumina). Reads were trimmed using Cutadapt v1.16.6 (8) and quality controlled using FastQC v0.72 (https://github.com/s-andrews/FastQC). High-quality sequence reads were assembled *de novo* using Unicycler v0.4.6.0 (9), which includes SPAdes v3.12.0 (10). Annotation was carried out using RASTtk v2.0 (11, 12) and the NCBI Prokaryotic Genome Annotation Pipeline (PGAP) v5.0 (13, 14). Phylogeny for exact identification was conducted with the codon tree pipeline in PATRIC v3.6.9 (15, 16), which uses single-copy cross-genus protein families and analyzes aligned proteins and coding DNA from single-copy genes using RAxML v8.0.0 (17). Default parameters were used for all software unless otherwise noted. Genome size and further information are presented in Table 1.

Antibiotic resistance genes were analyzed using the Comprehensive Antibiotic Resistance Database (CARD) v3.0.3 within PATRIC’s specialty gene table (18). Several multidrug-resistant efflux pumps (e.g., AcrAB, AcrAD, EmrAB, MacAB, and MdtABC) and antibiotic resistance genes (e.g., β-lactamase class C [*ampC*]) were found in all of the genomes. Homologues of the antimicrobial peptide resistance gene *dlt*, commonly known from Gram-positive bacteria (19), were present in the Enterobacter genomes.

Further analysis of the genomes of coliform bacteria from drinking water reservoirs will increase our knowledge about their hygienic relevance, their ecology, and their ability to proliferate in oligotrophic environments.

### Data availability.

The whole-genome shotgun projects and the raw sequence reads have been deposited at DDBJ/ENA/GenBank under the accession numbers listed in Table 1. They belong to BioProject PRJNA680915. For all sequences, the first versions are described in this paper.
